# Cerebrospinal Fluid‐Derived Extracellular Vesicles: A Proteomic and Transcriptomic Comparative Analysis of Enrichment Protocols

**DOI:** 10.1002/jex2.70076

**Published:** 2025-08-11

**Authors:** Marta García‐Arauzo, Sandrine Reymond, Lyssia Gruaz, Domitille Schvartz, Natacha Civic, Mylène Docquier, Christine Deffert, Pascal Colosetti, Jean‐Charles Sanchez, Claire Bridel

**Affiliations:** ^1^ Translational Biomarker Group, Department of Medicine, Faculty of Medicine University of Geneva Geneva Switzerland; ^2^ Proteomics Core Facility, Faculty of Medicine University of Geneva Geneva Switzerland; ^3^ Biostatistics Core Facility, Faculty of Medicine University of Geneva Geneva Switzerland; ^4^ iGE3 Genomics Platform, Faculty of Medicine University of Geneva Geneva Switzerland; ^5^ Division of Laboratory Medicine Geneva University Hospitals Geneva Switzerland; ^6^ Université Claude Bernard Lyon1 CarMeN Laboratory, Inserm, INRAe Lyon France; ^7^ Department of Clinical Neurosciences Geneva University Hospital Geneva Switzerland

**Keywords:** cerebrospinal fluid, exosomes, extracellular vesicles, mass spectrometry, RNA sequencing, size‐exclusion chromatography, ultracentrifugation

## Abstract

Proteomic and transcriptomic analyses of cerebrospinal fluid (CSF)‐derived extracellular vesicles (EVs) offer unique insights into molecular changes associated with central nervous system (CNS) diseases and may result in biomarker identification. No gold standard method to enrich EVs from CSF has been established, and head‐to‐head comparisons of outputs of different protocols are scarce. Using a large pool of CSF, we characterised the EV preparations resulting from four enrichment protocols and compared them in terms of yield and purity. We found that particles enriched by ultracentrifugation (UC) or a combination of ultrafiltration and size exclusion chromatography (UF‐SEC) exhibited the typical morphological and biochemical characteristics of small EVs and were highly enriched in proteins and polyadenylated (polyA) transcripts associated with EV‐related biological processes. UF‐SEC preparations had higher particle yields, whilst more proteins were identified in UC preparations. Approximately 40% of the EV preparations’ proteome was not identified in unenriched CSF, among which a core proteome of 45 proteins was identified in 30 EV preparations from independent experiments, which may serve as CSF‐derived EV markers. Enrichment scores to protein contaminants, albumin and apolipoprotein E were higher in UF‐SEC preparations. In conclusion, all protocols analysed here resulted in enrichment of particles with small EV characteristics, with EV enrichments from UF‐SEC resulting in the highest yield and purity.

## 1 Introduction

The extracellular vesicle (EV) proteome and transcriptome consist of a sample of that of the cell of origin (Martin‐Jaular et al. 2021; Bellingham et al. 2012). EVs have thus emerged as circulating molecular footprints of the cellular biological state, offering unique insights into disease‐associated molecular changes (Grapp et al. 2013; Schiera et al. 2015). EVs were first described in cerebrospinal fluid (CSF) 15 years ago, and research into their cellular origin suggests a significant proportion is released by central nervous system (CNS) cells, the remainder probably originating from immune cells within the CNS and CSF, choroid plexus epithelial cells, and peripheral tissues (Harrington et al. 2009; Chiasserini et al. 2014; Guha et al. 2019; Thompson et al. 2018; Balusu et al. 2016). Interest in CSF‐derived EVs as a source of molecular biomarkers of pathological conditions restricted to the CNS has grown steadily, and candidate molecular biomarkers for a variety of CNS conditions have been reported (Welton et al. 2017). Reproducibility remains, however, challenging, in part due to preanalytical and analytical variables that vary extensively between studies and are not always reported precisely (Jin et al. 2021). To address some of these limitations, the International Society for Extracellular Vesicles (ISEV) advocates a detailed and structured reporting of preanalytical and analytical methods for CSF‐derived EV enrichment (Sandau et al. 2024). No gold standard method to enrich EVs from CSF has been established, and head‐to‐head comparisons of outputs of different protocols are scarce (Kangas et al. 2023). This study aims at comparing EV preparations resulting from four enrichment protocols in terms of size, yield, and purity with respect to protein contaminants, using nanoparticle tracking analysis (NTA), transmission electron microscopy (TEM), western blotting, liquid chromatography tandem mass spectrometry (LC‐MS/MS), and polyadenylated (polyA) RNA sequencing (RNAseq) to characterise outputs. In a literature survey, we identified ultracentrifugation (UC) as the most widely used enrichment method for CSF‐derived EVs (Chiasserini et al. 2014; Stuendl et al. 2016; Vella et al. 2008; Harrington et al. 2009; Saman et al. 2012; Street et al. 2012; Patz et al. 2013; Geraci et al. 2018; Lee et al. 2016; Wang et al. 2017; Wang et al. 2017; Vacchi et al. 2021; Przybycien‐Szymanska et al. 2016; Manek et al. 2018; Kuharić et al. 2019; Li et al. 2022; Akers et al. 2013; Akers et al. 2015; Akers et al. 2016; Akers et al. 2016; Chen et al. 2013; Shi et al. 2015; Figueroa et al. 2017; He et al. 2019; Mohammadinasr et al. 2023; Caldi Gomes et al. 2021; Yagi et al. 2017; Emelyanov et al. 2020; Gu et al. 2021; Michel et al. 2022; da Cruz et al. 2020; Jain et al. 2019; Chatterjee et al. 2023; Gui et al. 2015; Ter‐Ovanesyan et al. 2021), followed by size‐exclusion chromatography (SEC) (Welton et al. 2017; Krušić Alić et al. 2022; Kurtjak et al. 2022; Hayashi et al. 2020; Kurzawa‐Akanbi et al. 2021; Thompson et al. 2020). A number of other studies used a variety of commercial affinity‐based methods, most of them consisting of magnetic beads coated with antibodies directed against tetraspanins CD9, CD63 and CD81. Both UC and SEC protocols are compatible with downstream LC‐MS/MS analyses and are unbiased, which is not the case of affinity‐based assays. For this reason, and because UC and SEC protocols are more amenable to optimisation than commercial assays, we focused this study on comparing these two enrichment methods. Using a large pool of CSF as starting material, we compared one UC protocol, two SEC protocols preceded by ultrafiltration (UF) for volume reduction, and a protocol combining UC and SEC (UC‐SEC). We found that particles enriched by UC and UF‐SEC exhibited the typical morphological and biochemical characteristics of small EVs and are enriched in proteins and polyA transcripts highly associated with EV‐related biological processes. The output of the UC‐SEC protocol was too low for downstream characterisation. UF‐SEC preparations resulted in higher particle yields, whilst more proteins were identified by LC‐MS/MS in UC preparations. Approximately 40% of the EV preparations’ proteome was not identified in unenriched CSF, among which a core proteome of 45 proteins was identified in 100% EV preparations originating from four independent experiments on different CSF pools. These proteins are thus candidate markers of CSF‐derived EVs. Enrichment scores to albumin, a major plasmatic contaminant, and to apolipoprotein E, a major CNS lipoprotein (LPP) contaminant, were highest in UF‐SEC35 preparations. Finally, we found that using the UF‐SEC35 enrichment protocol, CSF storage at −80°C prior to EV enrichment, or EV storage at −80°C did not affect particle yield as assessed by NTA.

## 2 Materials and Methods

### 2.1 CSF Collection and Storage

**FIGURE 1:**
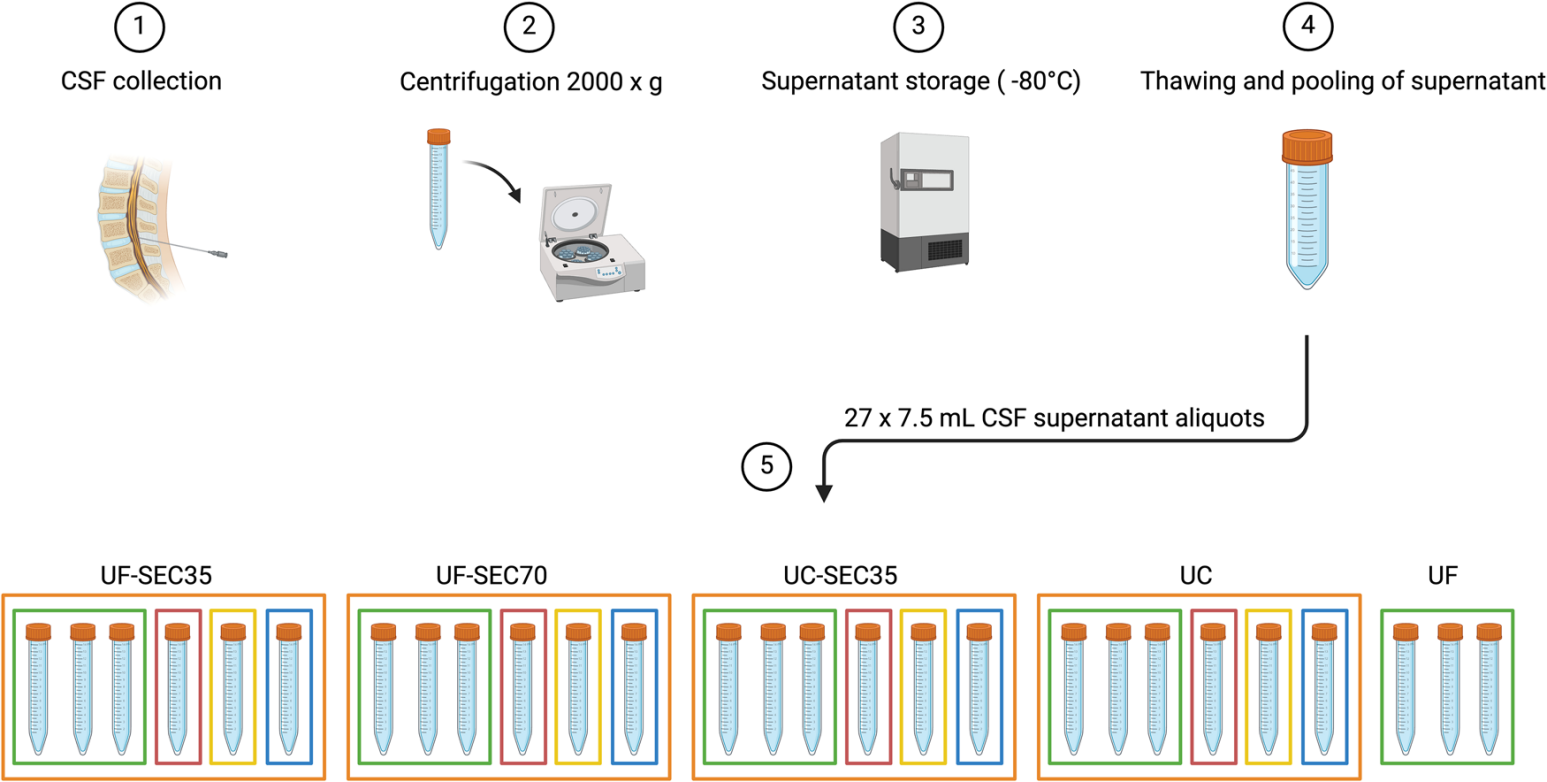
Experimental workflow. Cerebrospinal fluid (CSF) was collected by lumbar puncture (1), centrifuged (2), and the supernatant stored at −80°C until further use (3). CSF supernatant was thawed and pooled (4) and divided into 27 replicates of 7.5 mL on the day of extracellular vesicle (EV) enrichment (5). EVs were enriched with the UF‐SEC35 protocol (sextuplicates), the UF‐SEC70 protocol (sextuplicates), the UC‐SEC35 protocol (sextuplicates) and the UC protocol (sextuplicates). Triplicates were subjected to UF only. Nanotracking analysis was performed on all replicates enriched for EVs (orange). Triplicates were used for mass spectrometry (green), and single samples were used for RNA sequencing (pink), transmission electron microscopy (yellow) and western blotting (blue). SEC35, size exclusion chromatography with optimal recovery range of 35–350 nm; SEC70, size exclusion chromatography with optimal recovery range of 70–1000 nm; UC, ultracentrifugation; UF, ultrafiltration.

CSF was collected by lumbar puncture at the Geneva University Hospital with 22‐gauge needles. Anonymised left‐over material that was not needed for clinical analyses was used in this study, in accordance with the institution's ethical committee and national regulations. Non‐haemorrhagic CSF samples were selected and centrifuged at 2000 × *g*, 4°C for 20 min to pellet cells and cellular debris. Supernatant was frozen at −80°C until further use. On the day of EV enrichment, CSF supernatant from different anonymised sources was thawed, pooled and mixed, to obtain 205 mL homogeneous CSF supernatant. Twenty‐seven replicates of 7.5 mL CSF supernatant were prepared, and 2.5 mL CSF unenriched supernatant was kept aside as a control for LC‐MS/MS analyses and western blotting (Figure 1). We selected 7.5 mL as starting material because preliminary experiments indicated it was a volume allowing for robust downstream MS analysis.

### 2.2 UC

Twelve 7.5 mL replicates were centrifuged in a Beckman Coulter Optima XPN‐100 Ultracentrifuge with a SW55 Ti rotor at 4°C (Figure 1, UC and UC‐SEC35 replicates), first at 10,000 × *g* for 30 min to pellet cell debris. The supernatant was carefully collected and transferred to a new tube. Next, samples were centrifuged at 100,000 × *g* at 4°C for 120 min to pellet EVs. The supernatant was carefully removed, and crude EV pellets were resuspended in 1 mL ice‐cold PBS. A second round of UC 100,000 × *g* at 4°C for 120 min was carried out. Each resulting pellet was resuspended in 150 µL PBS. For the UC samples, 24 µL of each sample was used for NTA, which was performed immediately after enrichment, and the remainder 126 µL was stored at −80°C until further use. For the UC‐SEC35 samples, the samples (150 µL) were overlaid on SEC35 columns and processed as described below.

### 2.3 Size Exclusion Chromatography (SEC)

Fifteen 7.5 mL replicates were concentrated by UF to a final volume of 150 µL with 100 kDa Amicon Ultra‐15 centrifugal filters (Merck Millipore) and centrifugation at 2000 × *g*, 4°C, 30 min (Figure 1, UF‐SEC35, UF‐SEC70, UF). For the UF‐SEC35, UF‐SEC70, and UC‐SEC35 protocols (Figure 1), the ultrafiltrated or UC‐enriched samples were overlaid on SEC qEV columns (qEVoriginal 35 nm Gen 2 and qEVoriginal 70 nm Gen 2 from IZON Science LTD, respectively) following the manufacturer's instructions. For all samples, elution was performed with PBS, and ten 170 µL fractions were collected using an automatic fraction collector (IZON Science LTD). 24 µL of each fraction was used for NTA, which was performed immediately after enrichment. EVs were recovered in Fractions 6 and 7 in the UF‐SEC35, UF‐SEC70 and UC‐SEC35 protocols (Figure S1). The 2 EV‐containing fractions were pooled and, together with the UF samples, stored at −80°C until further use.

### 2.4 NTA

All replicates (Figure 1, outlined in orange) were analysed using a Particle Metrix ZetaView instrument (Particle Metrix GmbH, Inning, Germany) and corresponding ZetaView software (version 8.05.12 SP1), immediately after enrichment, to measure the concentration and size of particles, and to identify the SEC fractions with highest particle concentration in the UF‐SEC35, UF‐SEC70 and UC‐SEC35 protocols (Gardiner et al. 2013). Calibration beads were diluted in water 1:250.000 (v:v) to a final volume of 1 mL. Samples were diluted 1:50 in sterile PBS to a final volume of 1.2 mL. The cell was washed with PBS after each sample. In scatter mode, the instrument pre‐acquisition parameters were set to a sensitivity of 80, a frame rate of 30 frames per second and a shutter speed of 100. Post‐acquisition parameters were set to a minimum brightness of 30, trace length of 15 and area between 10 and 1000 pixels. For each sample, the particle count was measured at eleven positions, with one cycle of reading per position.

### 2.5 Western Blotting

All samples (consisting of all 10 fractions for the SEC protocols and the pellet for the UC protocol) were air‐dried (SpeedVac, ThermoFisher) immediately after enrichment. The samples were then resuspended in water and Laemmli buffer (4% SDS, 20% glycerol, 10% 2‐mercaptoethanol, 0.004% bromophenol blue and 0.125 M Tris‐HCl pH 6.8). After boiling for 10 min at 95°C, the samples were loaded on 12% acrylamide SDS‐PAGE gels. Controls consisted of 10 ng proteins from unenriched CSF supernatant, and xx ng cell lysate. Protein content was measured using the Bradford Reagent (ab119216). Electrophoresis was run at 150 V for 180 min. Transfer was performed on Immuno‐Blot nitrocellulose membranes for 75 min, 40 V. Membranes were stained with Ponceau Red to reveal the proteins and with water to remove the excess. The blocking buffer was made by dissolving milk powder (to 5% w/v) in PBS‐T (PBS with 0.1% Tween). Nitrocellulose membranes were blocked on a shaker overnight at 4°C, and then incubated with primary antibody for 3 h at room temperature (RT). Membranes were washed three times with PBS‐T and incubated with the corresponding secondary antibody for 1 h at RT. Antibodies and dilutions used are detailed in Table S1. Membranes were washed three times with PBS‐T, and SuperSignal West Pico PLUS Chemiluminescent Substrate (ThermoScientific) was added. Images were acquired with myECL Imager (ThermoScientific). Band intensities were quantified using ImageJ.

### 2.6 Electron Microscopy

The replicates for TEM (Figure 1, outlined in yellow) were air dried in a vacuum concentrator (SpeedVac, ThermoFisher), resuspended in 20 µL water and stored at 4°C until analysis. Suspensions were absorbed on 200‐Mesh Nickel grids coated with formvar‐C for 2 min at RT. After washing once in filtered distilled water, suspensions were coloured with 2% phosphotungstic acid for 2 min and examined using a Jeol transmission electron microscope JEM‐1400 (Jeol, Tokyo, Japan) equipped with a Gatan camera (Orius 1000) and Digital Micrograph Software.

### 2.7 Sample Preparation for Proteomic Analysis

Samples were thawed on ice and lysed with 0.1% RapiGest SF (Waters Corporation) in 0.1 M triethylammonium bicarbonate buffer (TEAB), pH 8.0. Samples were incubated for 10 min at 80°C, spun (14,000 g, 10 min) and the supernatant collected into a fresh tube. The supernatant was reduced with 5 mM Tris (2‐carboxyethyl) phosphine hydrochloride (TCEP), alkylated with 15 mM iodoacetamide, and digested with sequencing grade‐modified trypsin (Promega, 1:50 protease to protein ratio) overnight. The peptide mixture was then desalted with C18 spin columns (Harvard apparatus), dried under vacuum, re‐suspended in 5% acetonitrile, 0.1% formic acid and spiked with iRT peptides (Biognosys) 1:20. The totality of the sample was injected into the mass spectrometer. To estimate the protein concentration in samples, we performed an enrichment with each protocol in a separate experiment and used the output to measure protein concentration using a Bradford assay.

### 2.8 Data‐Independent Acquisition Mass Spectrometry (DIA MS) and Data Analysis

Samples were diluted in 10 µL of loading buffer (5% CH_3_CN, 0.1% FA), and 8 µL was injected on‐column. LC–ESI–MS/MS was performed on a Q‐Exactive HF Hybrid Quadrupole‐Orbitrap Mass Spectrometer (Thermo Fisher Scientific) equipped with an Easy nLC 1000 liquid chromatography system (Thermo Fisher Scientific). Peptides were trapped on an Acclaim pepmap100, C18, 3 µm, 75 µm × 20 mm nano trap‐column (Thermo Fisher Scientific) and separated on a 75 µm × 250 mm, C18, 2 µm, 100 Å Easy‐Spray column (Thermo Fisher Scientific). The analytical separation was run for 125 min using a gradient of H2O/FA 99.9%/0.1% (solvent A) and CH3CN/FA 99.9%/0.1% (solvent B). The gradient was run from 8% B to 28% B in 105 min, then to 42% B in 20 min, then to 95% B in 5 min with a final stay of 20 min at 95% B. The flow rate was 250 nL/min, and the total run time was 150 min. DIA was performed with MS1 full scan at a resolution of 60,000 (FWHM), followed by 30 DIA MS2 scan at a resolution of 30 (FWHM) with 28 m*/z* isolation width within an *m/z* range of 400–12,400. MS1 was performed with an AGC target of 3 × 10^6^ and a maximum injection time of 60 ms. DIA MS2 was performed using higher‐energy collisional dissociation (HCD) at 27%, an AGC target of 1 × 10^6^ and a maximum injection time of 50 ms.

DirectDIA analysis workflow was used in Spectronaut (Biognosys AG, Zurich, Switzerland) to match DIA MS raw data. Carbamidomethyl was defined as a fixed modification and oxidation of methionine as a variable modification. Protein and PSM false discovery rates were set to 0.01, and data filtering was set to *Q*‐value. The MS proteomics data have been deposited to the ProteomeXchange Consortium via the PRIDE partner repository with the dataset identifier **to be added**.

### 2.9 Core Proteome Definition

We defined the 100% and 80% core proteome as the proteins that were quantified in 100%, respectively 80%, of the samples across four independent experiments with different pools of CSF (Table S2).

### 2.10 Gene Ontology Enrichment Analysis of Proteomic and Transcriptomic Data Sets

Gene Ontologies term enrichment analysis, as well as associated plots, were performed with ClusterProfiler (REF) R package, using the EnrichGO function. Enrichment tests were calculated for GO terms, based on a hypergeometric distribution. P‐value cutoff was set to 0.05, and *Q*‐value cutoff to 0.01.

### 2.11 RNA Extraction

Total RNA was extracted using the qEV RNA Extraction Kit (RXT01, IZON Science LTD) according to the manufacturer's instructions. RNA separation, detection and quantitation were performed on a 2100 Bioanalyzer (Agilent Technologies).

### 2.12 RNAseq

Total RNA was extracted using the qEV RNA Extraction Kit (RXT01, IZON Science LTD) according to the manufacturer's instructions. RNA quality and quantity were assessed by a 2100 Bioanalyzer (Agilent Technologies) (Michel et al. 2022). RNA profiles showed the presence of a low molecular weight peak below 200 nucleotides, in accordance with the literature. Total RNA yield was similar in all three preparations (Table S5) and corresponded to 1.04–1.34 ng of RNA per mL of CSF supernatant starting material, similarly to what has been described in a study using large amounts of CSF supernatant as starting material (Michel et al. 2022).

The SMART‐Seq mRNA kit from Takara was used for reverse transcription and cDNA amplification according to the manufacturer's specifications, starting with 5 ng in average of RNA as input. 200 pg of cDNA were used for library preparation using the Nextera XT kit from Illumina. Library molarity and quality were assessed with the Qubit and Tapestation using a DNA High sensitivity chip (Agilent Technologies). Libraries were sequenced on a NovaSeq 6000 Illumina sequencer for 30 million SR100 reads. Libraries were sequenced on a NovaSeq 6000 Illumina sequencer for 30 M of SR100 reads.

Sequencing data are available on GEO with submission number GSE300627.

### 2.13 Statistical Analysis

**FIGURE 2:**
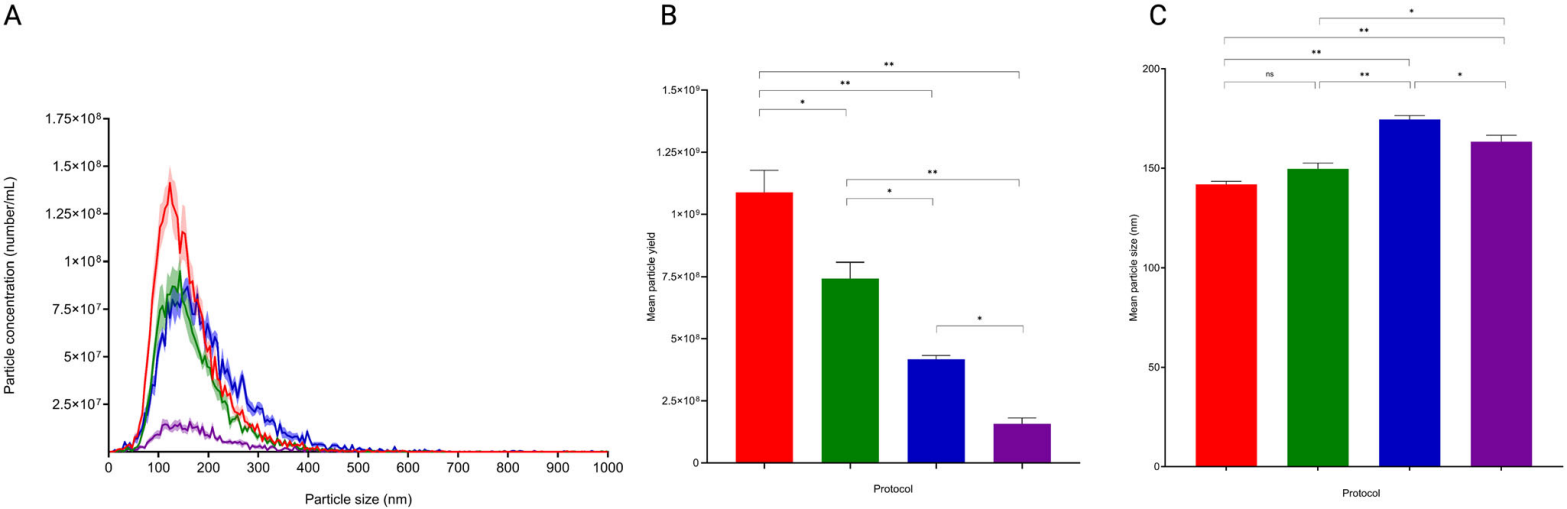
Nanotracking analysis characterisation of extracellular vesicle‐enriched samples. (A) Mean particle concentration per millilitre (mL) +/− standard deviation (SD, shade) by size in nanometres (nm) in sextuplicate extracellular vesicle (EV)‐enriched samples with UF‐SEC35 (red), UF‐SEC70 (green), UC (blue) and UC‐SEC35 (purple). (B) Mean particle yield (= particle concentration × total sample volume) and SD in sextuplicate EV‐enriched samples with UF‐SEC35 (red), UF‐SEC70 (green), UC (blue) and UC‐SEC35 (purple). (C) Mean particle size in nm and SD in sextuplicate EV‐enriched samples with UF‐SEC35 (red), UF‐SEC70 (green), UC (blue) and UC‐SEC35 (purple). SEC35, size exclusion chromatography with optimal recovery range of 35–350 nm; SEC370, size exclusion chromatography with optimal recovery range of 70–1000 nm; UC, ultracentrifugation; UF, ultrafiltration. *p < 0.05; **p < 0.01; ***p < 0.001.

Experiments reported in Figures 2 and S1 were performed in technical sextuplicates. Experiments reported in Figures 4, 5, and S2 were performed in technical triplicate. Comparisons between replicate means were performed using one‐way ANOVAs followed by Tukey post‐hoc tests in SPSS version 28.0.1.1. *p* values < 0.05 were considered statistically significant (*p < 0.05; **p < 0.01; ***p < 0.001).

## 3 Results

### 3.1 UF‐SEC, UC and UC‐SEC35 Protocols Enrich Particles With Mean Diameters in the Range of That of Small EVs

**FIGURE 3:**
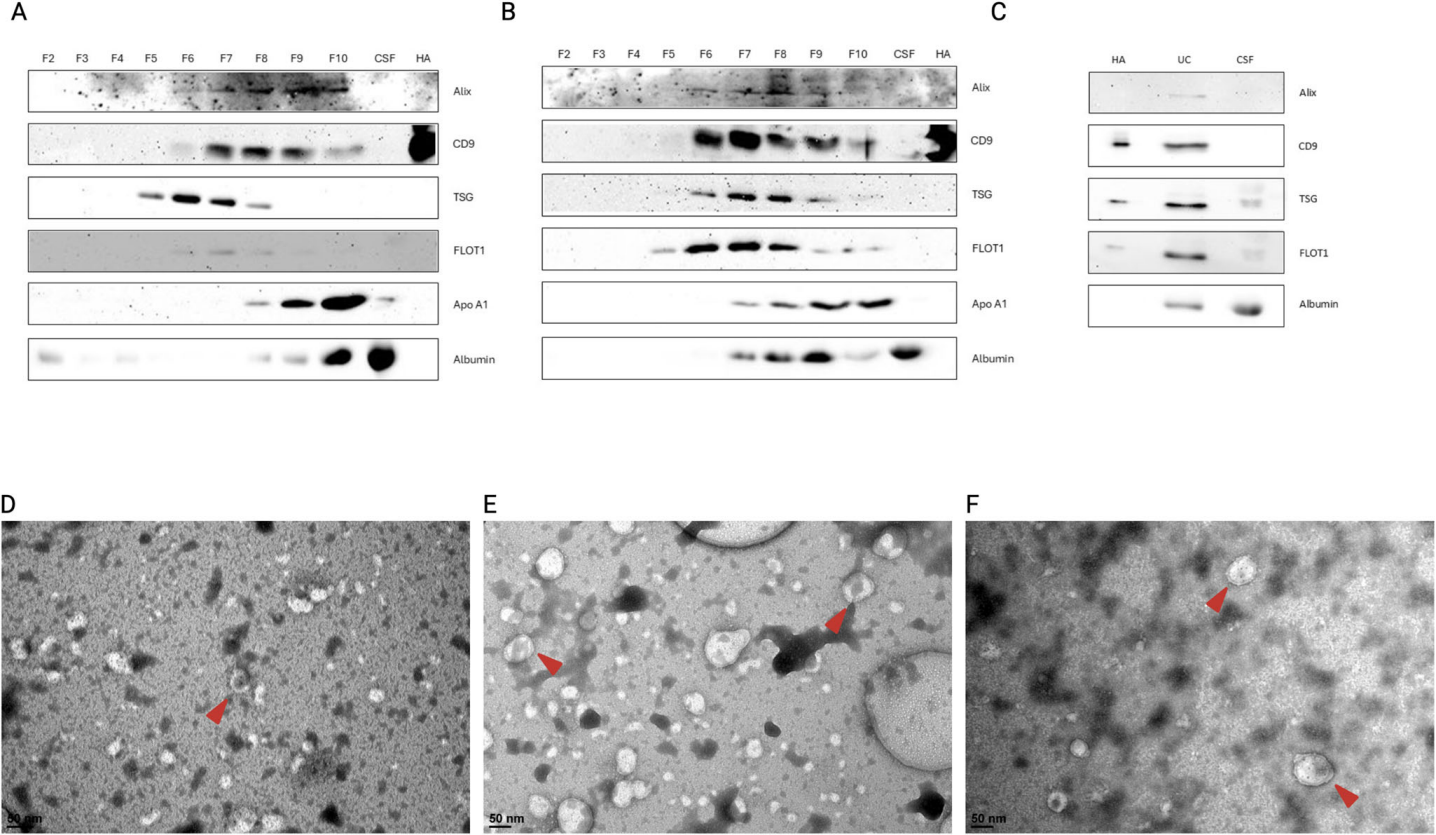
Western blot and transmission electron microscopy characterisation of extracellular vesicle‐enriched samples. (A) Western blot analysis of Alix, CD9, TSG101, FLOT1, ApoA1 and albumin in UF‐SEC35 fractions 2‐9, unenriched CSF and human primary astrocyte cellular lysate. (B) Western blot analysis of Alix, CD9, TSG101, FLOT1, ApoA1 and albumin in UF‐SEC70 fractions 2–9, unenriched CSF and human primary astrocyte cellular lysate. (C) Western blot analysis of Alix, CD9, TSG101, FLOT1 and albumin in UC pellet, cerebrospinal fluid, and human primary astrocyte cell lysate. (D– F) Representative transmission electron microscopy images of pooled fractions 6 and 7 of UF‐SEC35 (D) and UF‐SEC70 (E), and of UC pellet (F). Alix, ALG‐2‐interacting protein X; CD9, cluster of differentiation 9; CSF, cerebrospinal fluid; FLOT1, flotillin 1; TSG101, tumour susceptibility gene 101.

**FIGURE 4:**
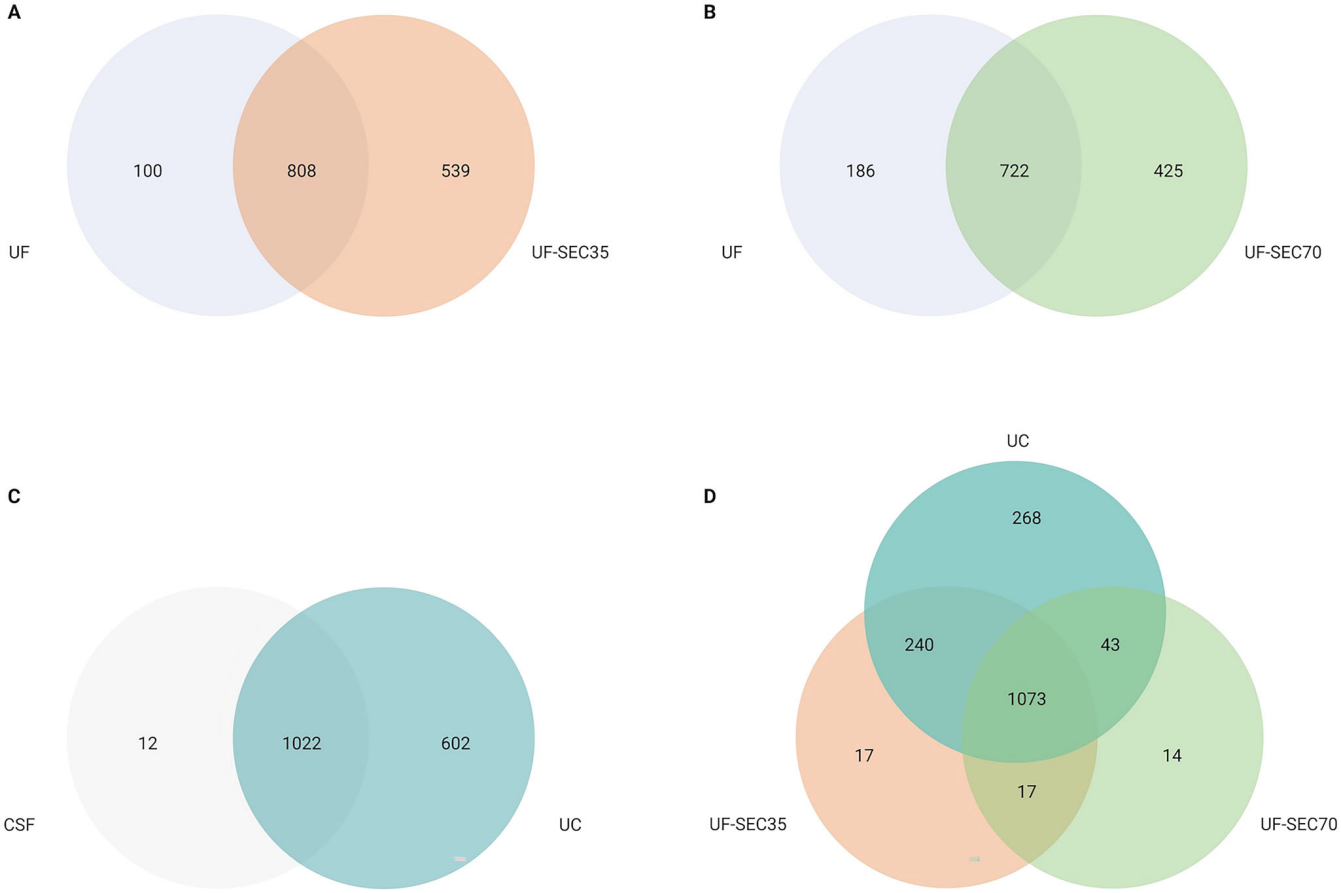
DIA‐MS analysis of extracellular vesicle‐enriched samples. (A) Venn diagram illustrating the overlap between proteins identified in triplicate samples enriched for extracellular vesicles (EV) with UF‐SEC35 (red), compared to those identified in triplicates subjected to UF only (purple). 37% of proteins (539/1447) are unique to the EV‐enriched samples. (B) Venn diagram illustrating the overlap between proteins identified in triplicate samples enriched for EVs with UF‐SEC70 (green), compared to those identified in triplicates subjected to UF only (purple). 32% of proteins (425/1333) are unique to the EV‐enriched samples. (C) Venn diagram illustrating the overlap between proteins identified in triplicate samples enriched for EVs with UC (turquoise), compared to those identified in triplicate CSF samples (orange). 37% of proteins (602/1636) are unique to the EV‐enriched samples. (D) Venn diagram illustrating the overlap between proteins identified in triplicate samples enriched for EVs with UF‐SEC35 (red), UF‐SEC70 (green) and UC (turquoise). 64% of proteins (1073/1672) are common to the UF‐SEC35, UF‐SEC70 and UC EV‐enriched samples, and 95% of proteins (1330/1404) are common to the UF‐SEC35 and UF‐SEC70 EV‐enriched samples. (E and F) Boxplots illustrating protein intensity measured by mass spectrometry in triplicates for EV‐enriched samples (UF‐SEC35, UF‐SEC70 and UC) and controls (UF, CSF). (E) Protein intensity of 20 proteins considered pan‐EV markers according to Exocarta database. (F) Protein intensity of 15 most abundant CSF proteins derived from plasma, which may be considered contaminants. (G) Enrichment scores (ES) calculated as mean intensity of EV marker divided by the mean albumin intensity in the same EV‐enriched sample. A2M, Alpha‐2‐Macroglobulin; ACTB, actin; ALB, albumin; ANXA2, annexin 2; APOA1, apolipoprotein A‐I; APOA2, apolipoprotein A‐II; APOB, apolipoprotein B; CD9, cluster of differentiation 9; CD47, cluster of differentiation 47; CD81, cluster of differentiation 81; EEF1A1, elongation factor 1 alpha 1 ; ENO1, alpha‐enolase; FLOT1, flotillin 1; FLOT2, flotillin 2; GAPDH, Glyceraldehyde‐3‐phosphate dehydrogenase; GC, vitamin D binding protein; HP, HP protein; HPX, hemopexin; HSP90AA1, heat shock protein HSP 90‐alpha; HSP90AB1, heat shock protein HSP 90‐beta ; HSPA8, heat shock cognate 71 kDa protein; IGHG1, Immunoglobulin heavy constant gamma 1; IGHG2, Immunoglobulin heavy constant gamma 2; ITGB1, Integrin beta‐1; LDHA, L‐lactate dehydrogenase A chain; ORM1, Alpha‐1‐acid glycoprotein 1; PDCD6IP, programmed cell death 6‐interacting protein; PKM, pyruvate kinase PKM ; SDCBP, Syntenin‐1 ; PTGDS, prostaglandin D2 synthase; SEC35, size exclusion chromatography with optimal recovery range of 35–350 nm; SEC37, size exclusion chromatography with optimal recovery range of 70–1000 nm;SERPINA1, alpha‐1 antitrypsin; TF, transferrin; TSG101, tumour susceptibility gene 101 protein ; TTR, transthyretin; UC, ultracentrifugation; UF, ultrafiltration; YWHAZ, 14‐3‐3 protein zeta/delta.

**FIGURE 5:**
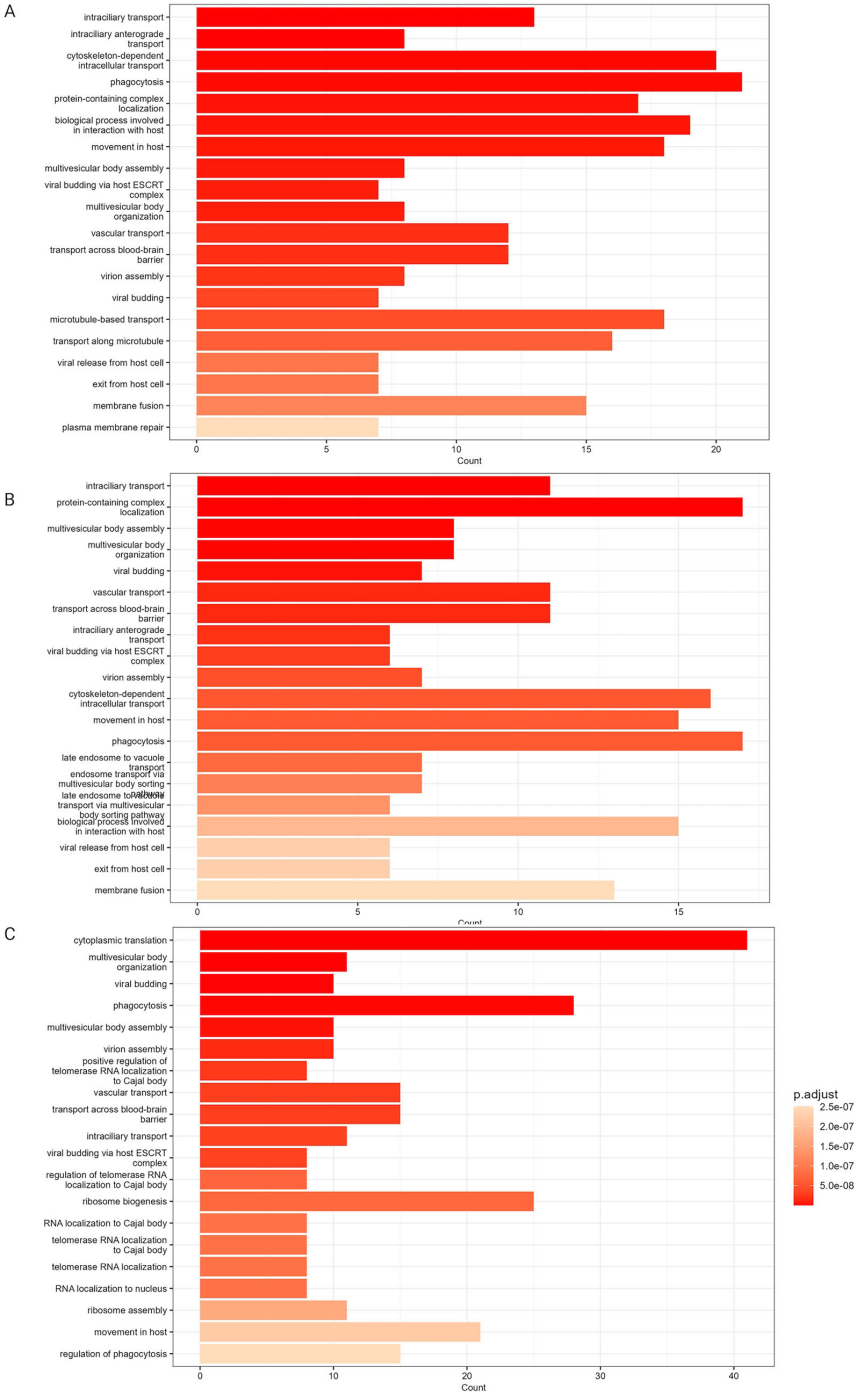
Biological processes associated with proteins unique to extracellular vesicle‐enriched samples. Biological processes associated with the proteins unique to triplicates enriched for extracellular vesicle (EV) with UF‐SEC35 (A), with UF‐SEC70 (B) and UC (C).

EVs were enriched from 7.5 mL pooled CSF supernatant in sextuplicate using four different protocols (Figure 1). Two protocols consisted of UF, to concentrate the starting volume of CSF from 7.5 mL to 150 µL, followed by SEC on agarose resin columns with either an optimal recovery range of particles with diameters between 35 and 350 nm (Figure 1, UF‐SEC35), or with an optimal recovery range of particles with diameters between 70 and 1000 nm (Figure 1, UF‐SEC70). We selected these two column types on the basis of the reported size of EVs in CSF (Sjoqvist et al. 2020). One protocol consisted of differential UC (Figure 1, UC), and one of differential UC followed by SEC35 (Figure 1, UC‐SEC35). NTA was used to measure the concentration and size of the particles in the ultracentrifuged pellets and each of the 10 fractions resulting from SEC. In the latter, Fractions 6 and 7 contained the highest particle concentration in all SEC protocols (Figure S1) and were pooled. Particle yield (i.e. the total amount of particles in each sample calculated as concentration of particles x total sample volume) was highest for the UF‐SEC35 protocol (Figure 2A,B, red), followed by the UF‐SEC70 protocol (Figure 2A,B, green), the UC protocol (Figure 2A,B, blue) and the UC‐SEC35 protocol (Figure 2A,B, purple), the latter containing too little material for downstream characterisation and proteomic analysis. Therefore, the results presented in Figures 3, 4, 5 do not include the output of the UC‐SEC35 protocol. Mean particle size was comparable in the UF‐SEC35 (Figure 2C, 142 ± 3.8 [SD] nm) and UF‐SEC70 (Figure 2C, 150 ± 7.3 [SD] nm) samples, but slightly higher in the protocols involving UC (Figure 2C, 174 ± 4.7 [SD] nm for the UC samples and 163 ± 7.9 [SD] nm for UC‐SEC35 samples). This size range is compatible with that of small EVs (Welsh et al. 2024).

### 3.2 Particles Enriched With UF‐SEC35, UF‐SEC70 and UC Exhibit Biochemical and Morphological Characteristics of EVs

The presence of pan‐EV markers was evaluated by western blotting of fractions 3–10 of SEC preparations and UC pellet. ALG‐2‐interacting protein X (ALIX), CD9, Tumour susceptibility gene 101 (TSG101) and flotillin 1 (FLOT1) were enriched in the UC pellet compared to unenriched CSF, whilst albumin was depleted in the UC pellet compared to CSF (Figure 3C). In the SEC samples, TSG101 and FLOT1 were enriched in Fractions 6 and 7, corresponding to the fractions with the highest particle concentration (Figure S1), whilst CD9 and ALIX were enriched in slightly later fractions, possibly indicating they are more abundant in smaller EVs (Figure 3A,B). In contrast, albumin and ApoA1, considered contaminants in the EV enrichment process, were enriched in Fractions 9 and 10, in which soluble proteins are expected. TEM showed the presence of particles with double‐layered membranes, which is a characteristic of EVs but not of non‐vesicular particles that bear single‐layered membranes, such as lipoproteins (Figure 3D,E,F **, red arrowheads**).

### 3.3 Proteins Unique to EV Preparations Are Associated With EV‐Related Biological Processes

LC‐MS/MS was performed on triplicate EV preparations resulting from UF‐SEC35, UF‐SEC70 and UC, as well as on ultrafiltrated CSF supernatant (UF) and unprocessed CSF supernatant (CSF), the latter two as controls for the SEC and UC protocols, respectively (Figure 1). LC‐MS/MS identified 1347 proteins in the EV preparations resulting from the UF‐SEC35 protocol, 539 (40%) of which were not identified in the UF control samples (Figure 4A). 1147 proteins were identified in the EV preparations resulting from the UF‐SEC70 protocol, 425 (37%) of which were not identified in UF control samples (Figure 4B). 1624 proteins were identified in the EV preparations resulting from the UC protocol, 602 (37%) of which were not identified in the CSF control samples (Figure 4C). 1073 proteins overlapped between the EV preparations enriched with the 3 protocols, and 1080 proteins between the EV preparations enriched with the SEC protocols (Figure 4D).

The proteins unique to EV preparations (i.e., not identified in UF or CSF samples) are particularly interesting because they may include markers of CSF‐enriched EVs. We refer to them onwards as “EV‐associated proteins.” We performed a GO enrichment analysis of EV‐associated proteins resulting from the preparations of all three protocols and found they were enriched for proteins highly associated with biological processes relevant to EVs. For all 3 protocols, intraciliary transport was one of the most significantly enriched biological processes (Figure 5). Primary cilia are immotile, mechanosensory organelles present on the surface of most human cells, highly enriched in receptors, ion channels and downstream effectors for several signalling pathways, playing an important role in cellular homeostasis (Hilgendorf et al. 2024). In addition, they were recently identified as hot spots of EV release (Luxmi and King 2022). Other highly enriched biological processes included multivesicular body assembly and the endosomal sorting complex required for transport pathway (ESCRT), which is involved in exosome formation (Gurung et al. 2021). These data suggest that a high proportion of EV‐associated proteins are indeed of EV origin.

**FIGURE 6:**
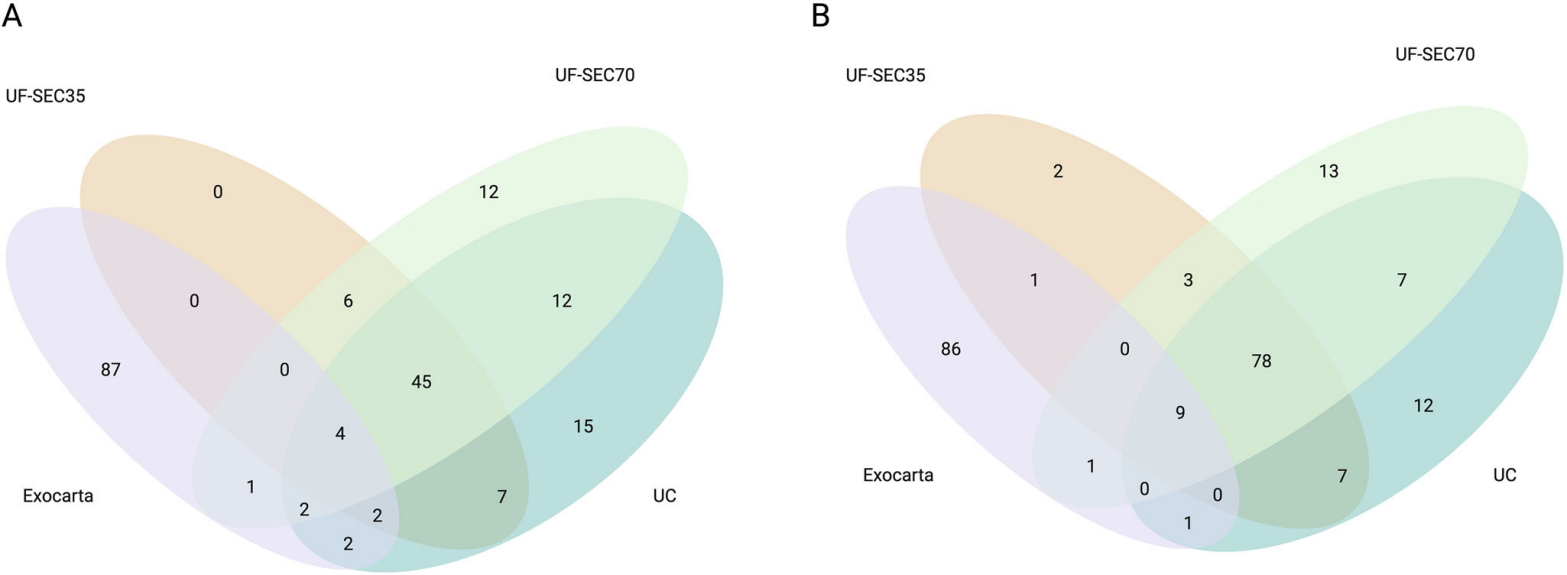
Core proteomes. Venn diagram illustrating the overlap between the core proteomes of samples enriched for extracellular vesicles (EVs) with UF‐SEC35, UF‐SEC70 or UC, and 20 pan‐EV markers from Exocarta. (A) 100% core proteome. (B) 80% core proteome.

To explore the potential of EV‐associated proteins to serve as markers of CSF‐derived EVs, we determined the 100% and 80% core proteome of these proteins, corresponding to the EV‐associated proteins identified in 100% CSF EV‐preparations or 80%, respectively, across independent experiments. To this end, we combined the present data (Table S2, manuscript data) with data from 3 additional proteomic experiments acquired in the laboratory over the past 2 years, using one or several of the enrichment protocols reported here, but critically, different CSF pools (Table S2, Experiments 1, 2 and 4). We found that 49 EV‐associated proteins were identified in 100% of the 30 replicates across four independent experiments (Figure 6A, 100% core proteome), and that 87 EV‐associated proteins were identified in 80% (Figure 6B, 80% core proteome). The 80% and 100% core proteomes are provided in Table S3. The 80% and 100% core proteomes do not include cell‐specific proteins. There is little overlap between the core proteomes and the top 100 proteins most frequently associated with EVs according to the ExoCarta database (Figure 6). However, the core proteomes include only those proteins that are exclusively identified in EV‐enriched samples, and not all those that are enriched in EV preparations compared to unenriched biofluids or cell culture medium.

### 3.4 EVs Enriched by UF‐SEC35 Have Higher Enrichment Scores to Albumin and Apolipoprotein E Than EVs Enriched by UF‐SEC70 or UC

**FIGURE 7:**
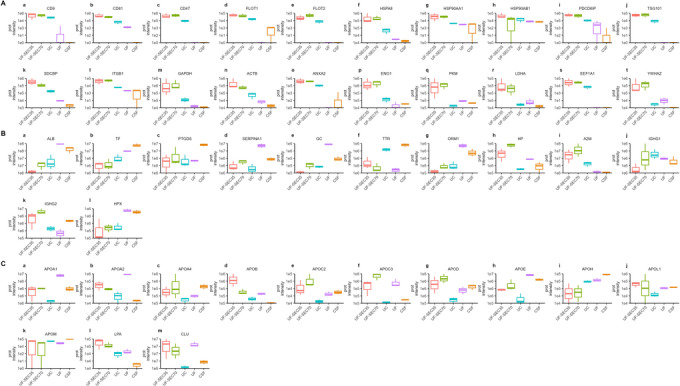
Median protein intensity of EV markers and contaminants across enrichment protocols and relevant control. (A) EV markers, (B) Plasmatic protein contaminants, (C) Apolipoproteins. Median intensity and interquartile range of triplicates are displayed for each protein.

Mean protein intensities of proteins considered pan‐EV markers, including actin (ACTB), annexin 2 (ANXA2), CD47, CD81, elongation factor 1 alpha 1 (EEF1A1), alpha‐enolase (ENO1), flotillin 1 (FLOT1), flotillin 2 (FLOT2), Glyceraldehyde‐3‐phosphate dehydrogenase (GAPDH), Heat shock protein HSP 90‐alpha (HSP90AA1), Heat shock cognate 71 kDa protein (HSPA8), Integrin beta‐1 (ITGB1), L‐lactate dehydrogenase A chain (LDHA), Programmed cell death 6‐interacting protein (PDCD6IP), Pyruvate kinase PKM  (PKM), Syntenin‐1 (SDCBP), Tumour susceptibility gene 101 protein  (TSG101) and 14‐3‐3 protein zeta/delta (YWHAZ) were higher in EV preparations by >1 log compared to UF and CSF control samples (Figure 7A). In contrast, mean protein intensities of most abundant CSF proteins of plasmatic origin including albumin (ALB), vitamin D binding protein (GC), hemopexin (HPX), Alpha‐1‐acid glycoprotein 1 (ORM1), alpha‐1 antitrypsin (SERPINA1) and transferrin (TF) were depleted by >1 log in EV preparations compared to UF and CSF control samples (Figure 7B). Other highly abundant CSF proteins of plasmatic origin, including alpha‐2‐Macroglobulin (A2M) and HP protein (HP) were higher in EV preparations by >1 log compared to UF and CSF control samples (Figure 7B), suggesting these plasmatic proteins are associated with EVs, possibly as part of the protein corona. Apolipoproteins (APO) are abundant in CSF, either originating from plasma (APOA1) or produced within the CNS (APOE) (Tsujita et al. 2024). They may be soluble, assembled in LPPs, or bound to EVs as part of their corona. We found that EV preparations were depleted in some apolipoproteins (APOA1, APOA2, APOE, APOH, Clusterin) whilst enriched in others (APOA4, APOB, APOC2, APOC3, APOD, lipoprotein (a))(Figure 7C). To compare enrichment performance between the three protocols with respect to plasma‐derived proteins, we calculated albumin enrichment scores (albES) and APOE enrichment scores (apoES) by dividing the mean protein intensities of each pan‐EV marker by that of albumin, respectively, APOE, in the same sample. Mean albES and mean apoES were higher in EV preparations enriched with UF‐SEC35 compared to the other protocols, suggesting the lowest contamination with plasma proteins and LPPs in EV preparations resulting from this protocol (Table S3A,B).

### 3.5 PolyA Transcriptome of EV Preparations Is Enriched for Transcripts Associated With EV‐Related Biological Processes

**FIGURE 8:**
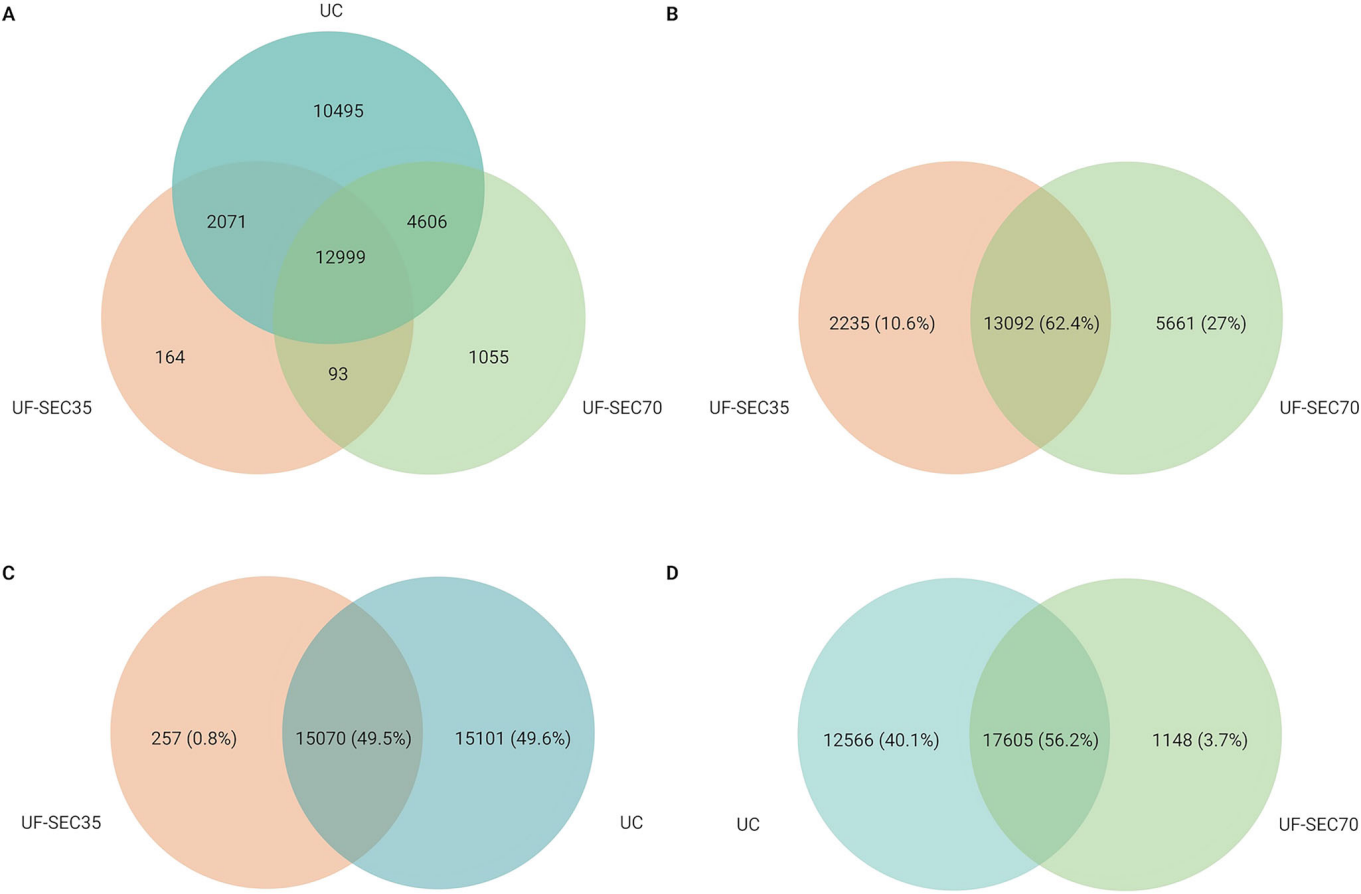
PolyA RNA seq of extracellular vesicle‐enriched samples. (A) Venn diagram illustrating the overlap between transcripts identified in samples enriched for extracellular vesicles (EV) with UF‐SEC35 (orange), UC (blue) and UF‐SEC70 (green). (B) Venn diagram illustrating the overlap between transcripts identified in samples enriched for EVs with UF‐SEC35 (orange) and UF‐SEC70 (green). (C) Venn diagram illustrating the overlap between transcripts identified in samples enriched for EVs with UF‐SEC35 (orange) and UC (blue). (D) Venn diagram illustrating the overlap between transcripts identified in samples enriched for EVs with UF‐SEC70 (green) and UC (blue).

**FIGURE 9:**
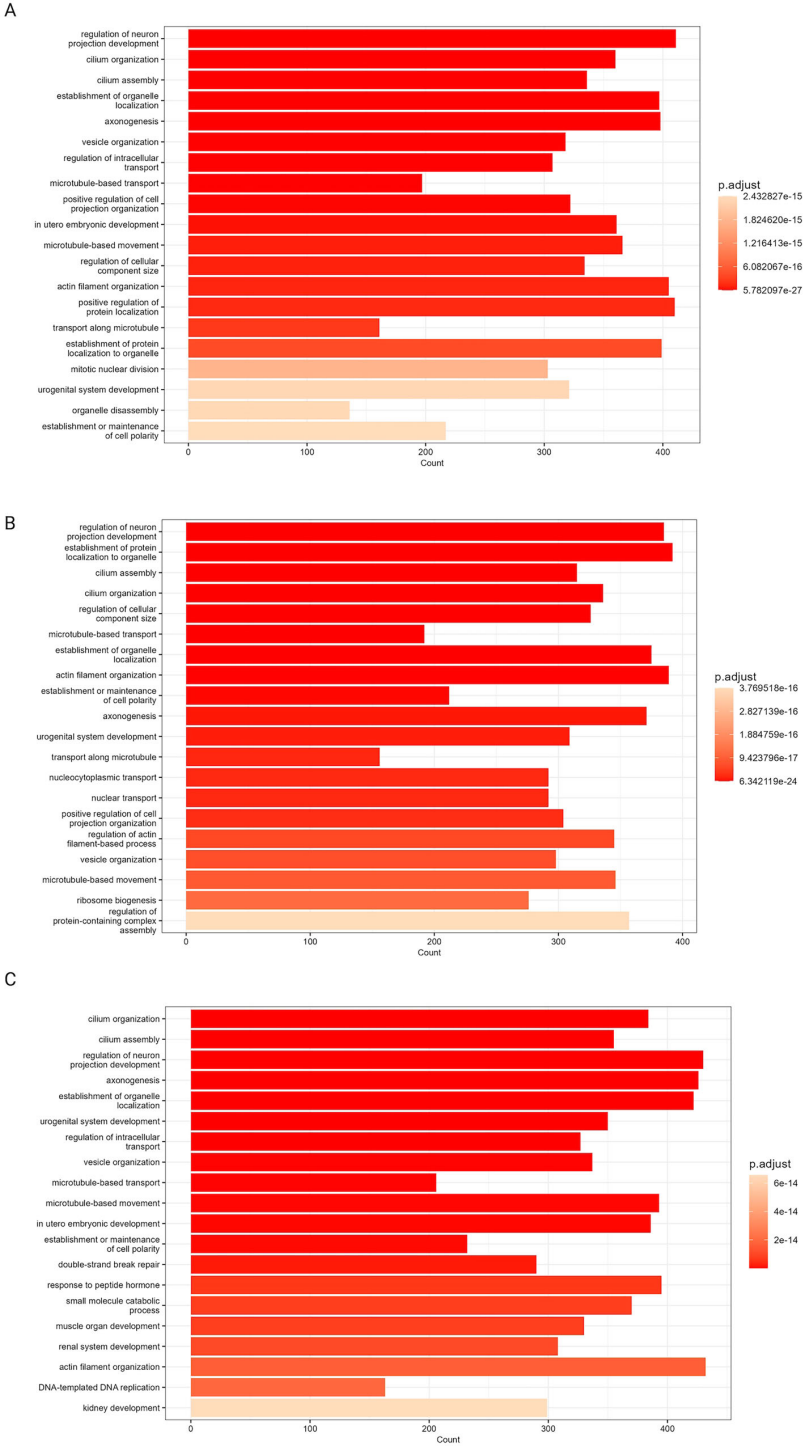
Biological processes associated with polyA transcripts in extracellular vesicle‐enriched samples. Biological processes associated with the transcripts identified in the output of UF‐SEC35 (A), with UF‐SEC70 (B) and UC (C).

We used polyA RNA‐seq, which comprehensively quantifies messenger RNAs (mRNAs) and long non‐coding RNAs (lncRNAs) to characterise and compare EV preparations’ transcriptome. RNA gel analysis showed the presence of low molecular weight bands below 200 nucleotides, also in accordance with the literature (Michel et al. 2022, Otake et al. 2019). Read counts were similar in all 3 protocols (3.3*10^6^ for the UF‐SEC35 EV preparation, 3.4*10^6^ for the UF‐SEC and UC EV preparations). Of these reads, more than 90% survived after adaptor trimming. For the UF‐SEC35 and UF‐SEC70 EV preparations, 59.2 % (1.9*10^6^) and 59.7% (2.0*10^6^) of the surviving reads were uniquely mapped to the human genome, respectively, whereas this proportion was significantly higher in the UC EV preparation (86.9%, 2.9*10^6^). Of the uniquely mapped reads, the number of genes detected with ≥10 counts was 22,440 (UF‐SEC35), 18,753 (UF‐SEC70) and 30,171 (UC), respectively (Table S5). Of these, 12,999 genes overlapped between the three protocols (Figure 8A), 13,092 genes (62.4%) between the UF‐SEC35 and UF‐SEC70 EV preparations (Figure 8B), 15,070 (49.5%) between the UF‐SEC35 and UC EV preparations (Figure 8C), and 17,605 (56.2%) between the UF‐SEC70 and UC EV preparations (Figure 8D). For all three protocols, the highest proportion of uniquely mapped genes with ≥10 counts was protein coding, the remainder consisting of lncRNAs. As expected, RNA species (ribosomal RNA, microRNAs) were rare, given the type of sequencing protocol chosen (Table S5). Cellular component GO enrichment analysis of the protein coding genes with >10 counts showed that these were enriched for genes associated with EV‐related biological processes (Figure 9A‐C), very similar to those identified on a protein level (Figure 5).

### 3.6 Effect of CSF Supernatant Storage and EV Preparation Storage on Particle Yield Measured by NTA

We performed a time‐course experiment using the UF‐SEC35 enrichment protocol to test the effect of CSF supernatant storage prior to enrichment on particle concentration. CSF was collected at time 0 (T0), centrifuged, pooled and 15 aliquots of 7.5 mL were prepared. Three aliquots were frozen at −80°C (Figure S2A,B, freeze thaw) and 3 were used to enrich EVs immediately (Figure 2A,B, T0). The remaining nine aliquots were kept at 4°C and EVs enriched after 24 h, 3 days and 7 days respectively (Figure 2A,B, D1, D3, D7). No difference in mean particle concentration as measured by NTA was observed between stored samples and samples processed on the day of CSF collection.

Next, we evaluated the effect of freezing EV preparations at −80°C immediately after enrichment on particle concentration. CSF was collected at T0, centrifuged and EVs enriched from 3 aliquots of 7.5 mL. NTA was performed immediately and repeated after storage of EV preparations for 7 days at −80°C (Figure 2C,D). No difference in particle concentration as measured by NTA was observed between fresh EV preparations and after a freeze‐thaw cycle (Figure 2C,D).

## 4 Discussion

Analysis of CSF‐enriched EV cargo offers unique insights into molecular changes and pathological pathways associated with CNS diseases (Malhotra et al. 2023). Additionally, CSF‐enriched EVs may be a source of molecular biomarkers of pathological processes restricted to the CNS, an organ not easily amenable to biopsy (Spinelli et al. 2025). EV enrichment from CSF is challenging because of the scarcity of these particles and the biological complexity of the matrix (Sandau et al. 2024). Pre‐analytical and analytical variables may affect EV yield, purity and integrity, challenging reproducibility (Sandau et al. 2024). The objective of this study was to compare the output of four enrichment protocols based on the two most employed biochemical EV enrichment methods identified in a literature survey. With a large pool of CSF supernatant as starting material, we performed a head‐to‐head comparison of EV preparations resulting from a UC protocol, two SEC protocols (UF‐SEC35 and UF‐SEC70) and a protocol combining UC and SEC (UC‐SEC35).

The characterisation of EV preparations is challenged by the lack of universal EV identification methods and molecular markers (Welsh et al. 2024). The ISEV recommends using multiple analytical methods to fulfill different EV characterisation requirements (Sandau et al. 2024, Welsh et al. 2024). Applying NTA, we found that particle size distribution was right‐skewed in all EV preparations, similarly to what is reported for plasma‐derived EVs, and that mean particle size was in the range of that of small EVs (Welsh et al. 2024, Holcar et al. 2020). Particles enriched with UC and a combination of UC and SEC35 had marginally higher mean sizes than those enriched with SEC, possibly related to particle aggregation during UC (Linares et al. 2015). Particle yield was highest with the SEC protocols, and lower with the UC protocol. We hypothesised that a combination of UC and SEC leads to higher EV purity by combining sedimentation rate‐based and size‐based separation techniques. However, particle yield with the UC‐SEC35 protocol was too low to allow for downstream proteomic analyses, and we focused on the comparison of EV preparations resulting from the UF‐SEC35, UF‐SEC70 and UC protocols. Western blot analysis of the protein content of SEC fractions showed that transmembrane (CD9), lipid raft‐associated (ALIX) and intracellular (TSG101) proteins, universally enriched in EV preparations (Keerthikumar et al. 2016), were detectable in particle‐containing fractions whilst depleted in non‐particle‐containing fractions or unenriched CSF (Kowal et al. 2016). In the output of the UC protocol (pellet), these proteins were detectable in the EV preparation and not in the same total protein content of unenriched CSF. LC‐MS/MS revealed that EV preparations were enriched for other proteins considered pan‐EV markers, compared to unenriched CSF supernatant or UF‐CSF (Keerthikumar et al. 2016, Kowal et al. 2016). About 37%–40% of the proteins identified by LC‐MS/MS were unique to EV preparations (i.e., not identified in unenriched CSF supernatant or UF‐CSF). We found that these proteins were enriched for proteins associated with EV formation, interciliary processes and clathrin. Compellingly, the transcripts identified in the EV preparations were also highly enriched in transcripts associated with cilium organisation and assembly. Applying TEM to EV preparations, we could visualise nanoparticles with characteristic lipid bilayer of EVs, a feature absent in non‐vesicular extracellular particles such as LPPs, which harbour a single phospholipid layer (Tsujita et al. 2024). Together, these results converge to indicate EVs are indeed enriched in the output of all three protocols studied here.

CSF is a paucicellular biofluid mostly produced in the lateral brain ventricles by the choroid plexus. Na^+^ transport across its epithelium generates osmotic gradients resulting in water and solute movement from plasma into the ventricular space (Wichmann et al. 2021). Contaminants in the EV enrichment process include plasmatic proteins, which are the most abundant proteins identified in CSF, and non‐vesicular EVs including LPPs, originating from plasma or produced within the CNS (Schilde et al. 2018, Tsujita et al. 2024). LPPs are about a 100 times less abundant in CSF than in plasma, and their physicochemical features resemble plasma HDL, with a size range smaller than that of EVs (<30 nm) (Tsujita et al. 2024, Jeppesen et al. 2023). We compared EV purity with respect to plasmatic protein albumin and APOE, one the most abundant CSF apolipoproteins, by calculating ES (albES and apoES) (Tsujita et al. 2024). We found that mean albES and mean apoE were highest in the EV preparations resulting from the UF‐SEC35 protocol and lowest in EV preparations resulting from UC, indicating the former protocol achieves better specificity. However, this strategy to assess EV purity has limitations, because it has recently been demonstrated that plasmatic proteins, in particular apolipoproteins, can be part of the EV corona, therefore co‐enriching with EVs without being contaminants (Tóth et al. 2021, Tóth et al. 2021).

In order to identify potential protein markers of CSF‐derived EVs, we identified a core proteome consisting of EV‐associated proteins identified in 100% and 80%, respectively, of EV preparations across four independent experiments performed on different CSF supernatant pools. We did not find any cell‐type‐specific proteins in either core proteomes. Validation studies are needed in other laboratories to confirm the potential of these proteins as markers of CSF‐derived EVs.

Finally, we addressed the question of the effect of CSF supernatant storage or CSF‐derived EV storage on particle counts as measured with NTA. We found that freezing CSF supernatant at −80°C prior to EV enrichment did not affect particle yield. Storage of CSF‐derived EVs at 4°C for up to a week did not affect particle yield either. These results are, however, preliminary as NTA does not allow for differentiation of EVs from non‐vesicular particle and protein aggregates, as discussed above. Nevertheless, it gives us a first indication that these pre‐analytical procedures do not drastically affect EV yield.

The CSF starting volumes used in this study were deliberately high in order to assure sufficient material for downstream proteomic and transcriptomic analyses. Aware that these volumes exceed what is usually available in clinical practice or biobanks, further studies are needed to determine the minimum volume needed to achieve robust results.

In conclusion, using a large volume of high‐quality CSF supernatant, we find that the EV output is significantly modified both in terms of proteome and transcriptome by the choice of enrichment protocol. SEC‐based protocols achieve better purity compared to a UC‐based protocol. The output of all protocols is highly enriched for proteins and transcripts associated with biological processes relevant to EVs, indicating an enrichment in EVs.

## Author Contributions

**Marta García‐Arauzo**: conceptualization, data curation, formal analysis, investigation, methodology, writing – review and editing. **Sandrine Reymond**: formal analysis, investigation, methodology, writing – review and editing. **Lyssia Gruaz**: data curation, formal analysis, investigation. **Domitille Schvartz**: formal analysis, writing – review and editing. **Natacha Civic**: data curation, formal analysis, writing – review and editing. **Mylène Docquier**: data curation, supervision, writing – review and editing. **Christine Deffert**: resources, writing – review and editing. **Pascal Colosetti**: formal analysis, resources, writing – review and editing. **Jean‐Charles Sanchez**: conceptualization, writing – review and editing. **Claire Bridel**: conceptualization, data curation, formal analysis, funding acquisition, investigation, methodology, project administration, writing – original draft.

## Conflicts of Interest

The authors declare no conflicts of interest.

## Supporting information




**Supplementary Figure 1** Mean particle concentration measured by NTA in fractions 5 to 8 of SEC protocols. Fraction 6 (F6) and fraction 7 (F7) consistently contain highest particle concentrations across sextuplicates in the output of the UF‐SEC35 and UF‐SEC70 protocols. In the UC‐SEC35 output, particle yield is lower and the fractions with highest particle concentrations vary across replicates. Concentration is number of particles per mL.

**Supporting Figure 2** Effect of storage and a freeze thaw cycle on EV enrichment or stability. **A** Effect of cerebrospinal fluid supernatant storage prior to EV enrichment on particle yield as assessed by NTA. **B** Effect of a freeze‐thaw cycle of EV preparations on particle concentration as assessed by NTA.

**Supporting Table 1** Antibodies and dilutions used for Western blot analysis

**Supporting Table 2** Four DIA‐MS experiments with different CSF pools as starting material for EV enrichment

**Supporting Table 3** 80% and 100 % core proteomes

**Supporting Table 4** Enrichment scores to albumin (**A**) and apolipoprotein E (**B**)

**Supporting Table 5** RNA species identified in the output of the 3 enrichment protocols.

**Supporting Table 6** Summary of the output of UF‐SEC35, UF‐SEC70, et UC enrichment protocols

## Data Availability

Data is available upon request to the corresponding author. Sequencing data are available on GEO with submission number GSE300627. Proteomic data are available on PRIDE with submission number xxx.
