# Supplementary figures and images for: Cerebrospinal Fluid‐Derived Extracellular Vesicles: A Proteomic and Transcriptomic Comparative Analysis of Enrichment Protocols
Source: J Extracell Biol. 2025 Aug 11;4(8):e70076. doi: 10.1002/jex2.70076 (PMC12339045; doi:10.1002/jex2.70076)

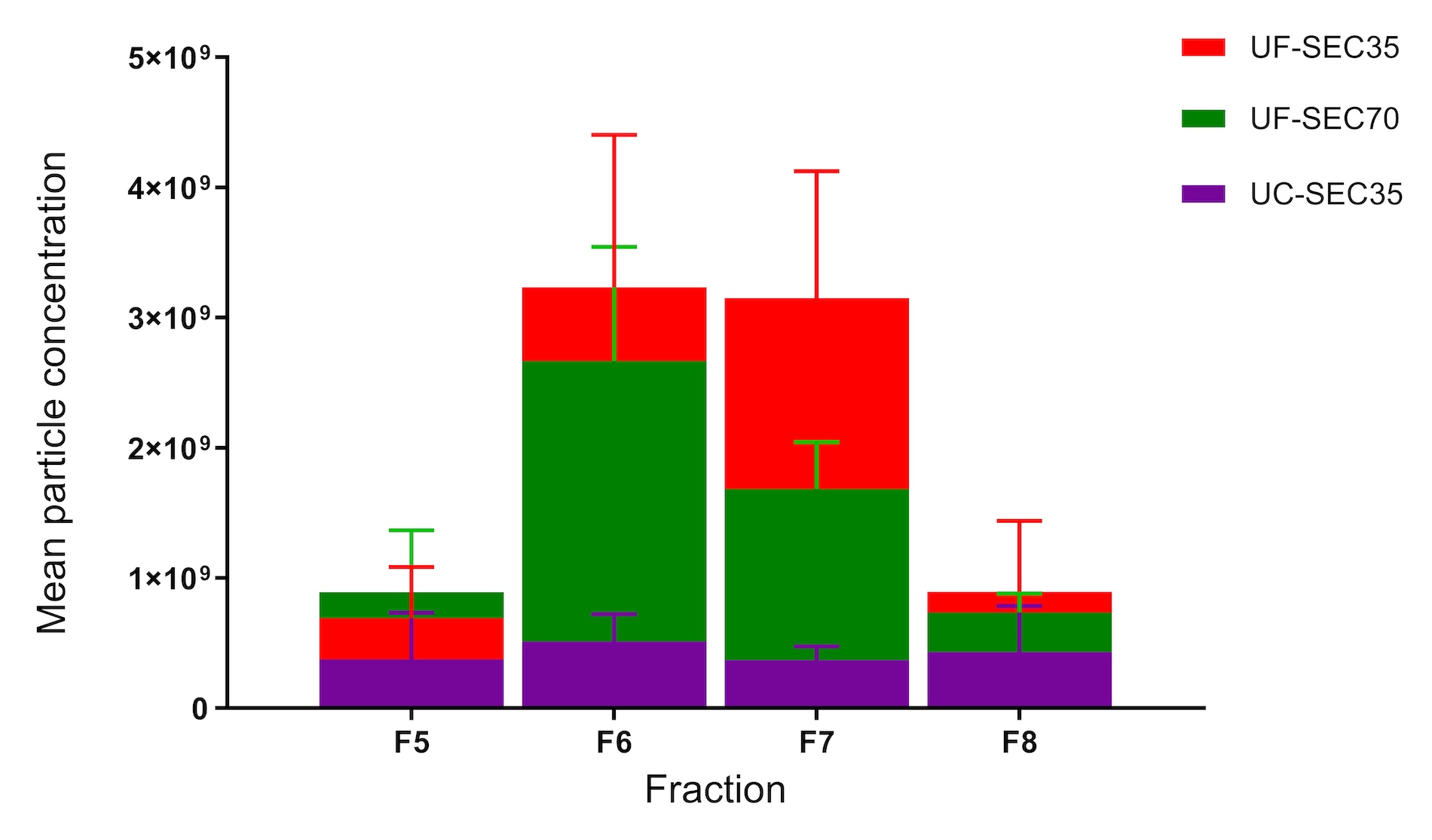

Supplement: Supplementary file 1 — Supplementary Figure 1 Mean particle concentration measured by NTA in fractions 5 to 8 of SEC protocols. Fraction 6 (F6) and fraction 7 (F7) consistently contain highest particle concentrations across sextuplicates in the output of the UF‐SEC35 and UF‐SEC70 protocols. In the UC‐SEC35 output, particle yield is lower and the fractions with highest particle concentrations vary across replicates. Concentration is number of particles per mL. [file JEX2-4-e70076-s001.jpg]

A

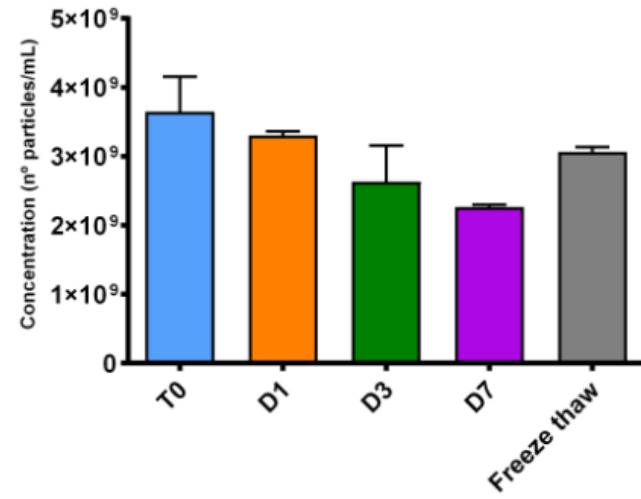

B

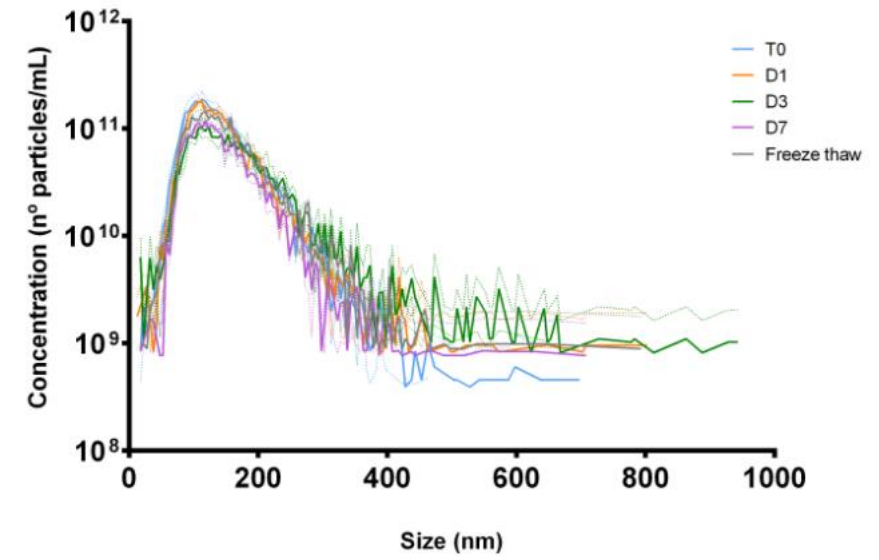

C

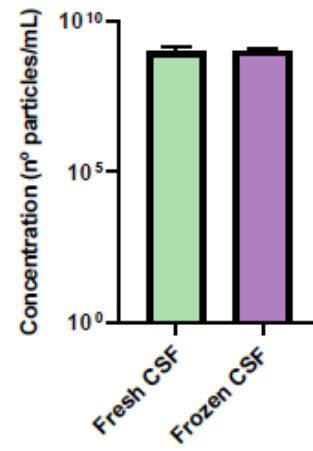

D

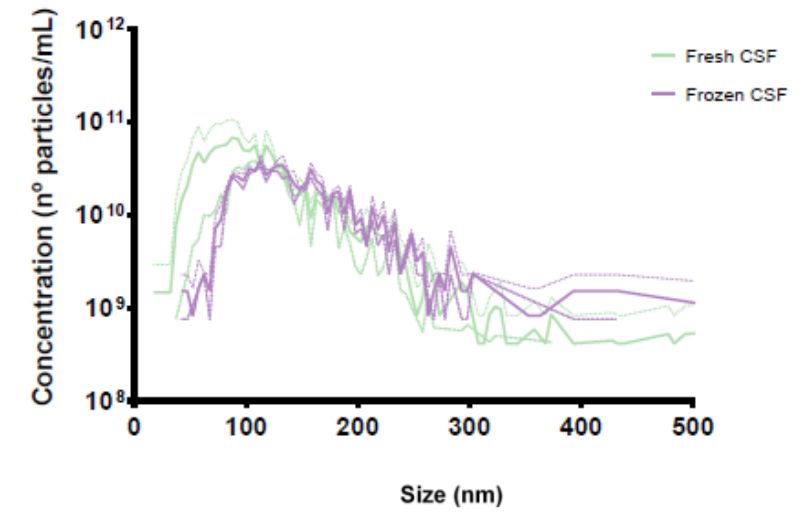

Supplement: Supplementary file 2 — Supporting Figure 2 Effect of storage and a freeze thaw cycle on EV enrichment or stability. A Effect of cerebrospinal fluid supernatant storage prior to EV enrichment on particle yield as assessed by NTA. B Effect of a freeze‐thaw cycle of EV preparations on particle concentration as assessed by NTA. [file JEX2-4-e70076-s005.pdf]

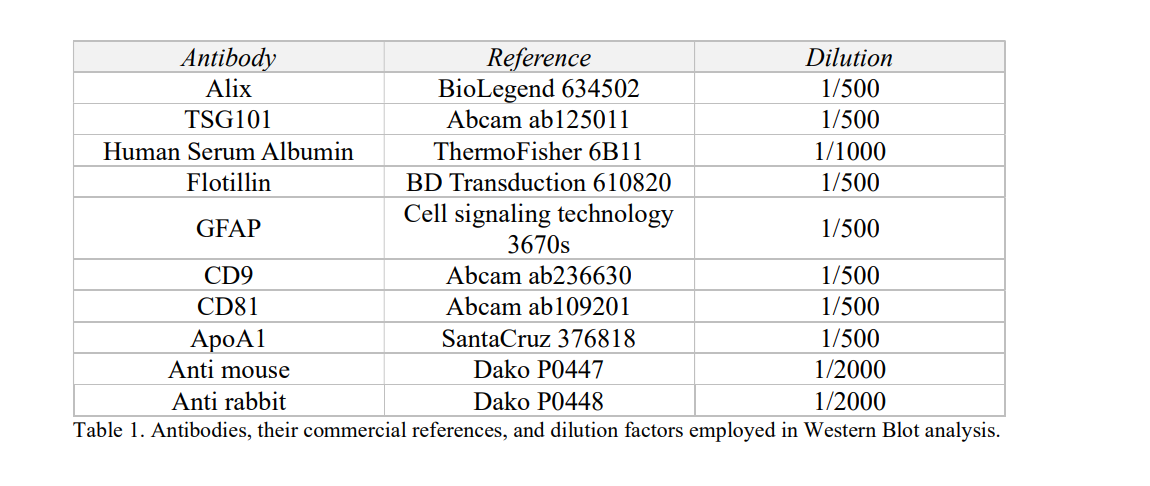

Supplement: Supplementary file 3 — Supporting Table 1 Antibodies and dilutions used for Western blot analysis [file JEX2-4-e70076-s007.docx]
