# Supplementary material for: Cerebrospinal Fluid‐Derived Extracellular Vesicles: A Proteomic and Transcriptomic Comparative Analysis of Enrichment Protocols
Source: J Extracell Biol. 2025 Aug 11;4(8):e70076. doi: 10.1002/jex2.70076 (PMC12339045; doi:10.1002/jex2.70076)
Supplement: Supplementary file 4 — Supporting Table 2 Four DIA‐MS experiments with different CSF pools as starting material for EV enrichment [file JEX2-4-e70076-s004.pdf]

	Experiment 3<br>(presented in manuscript)	Experiment 1	Experiment 2	Experiment 4
UF-SEC35 replicates	3	3	3	6
UF-SEC70 replicates	3	0	3	0
UC replicates	3	3	3	0
CSF starting volume per replicate in mL	7.5	10	10	3x 10 and 3x7.5
