# Supplementary material for: Cerebrospinal Fluid‐Derived Extracellular Vesicles: A Proteomic and Transcriptomic Comparative Analysis of Enrichment Protocols
Source: J Extracell Biol. 2025 Aug 11;4(8):e70076. doi: 10.1002/jex2.70076 (PMC12339045; doi:10.1002/jex2.70076)
Supplement: Supplementary file 6 — Supporting Table 4 Enrichment scores to albumin (A) and apolipoprotein E (B) [file JEX2-4-e70076-s003.docx]

**Supplementary Table 4**

**A**

| **EV marker** | **UF-SEC35 albES** | **UF-SEC70 albES** | **UC albES** |
| --- | --- | --- | --- |
| ACTB | 233 | 40 | 6 |
| ANXA2 | 336 | 78 | 5 |
| CD9 | 180 | 49 | 9 |
| CD47 | 217 | 84 | 3 |
| CD81 | 239 | 37 | 2 |
| EEF1A1 | 26 | 6 | 0 |
| ENO1 | 262 | 99 | 4 |
| FLOT1 | 67 | 19 | 2 |
| FLOT2 | 24 | 24 | 1 |
| GAPDH | 234 | 92 | 6 |
| HSPA8 | 183 | 62 | 4 |
| HSP90AA1 | 71 | 14 | 0 |
| HSP90AB1 | 21 | 3 | 1 |
| ITGB1 | 62 | 20 | 1 |
| LDHA | 63 | 19 | 3 |
| PDCD6IP | 236 | 70 | 3 |
| PKM | 223 | 82 | 4 |
| SDCBP | 585 | 64 | 6 |
| TSG101 | 30 | 13 | 1 |
| YWHAZ | 78 | 31 | 2 |
| **Mean albES (SD)** | **114 (139)** | **32 (32)** | **3 (2)** |

**B**

| **EV marker** | **UF-SEC35 apoES** | **UF-SEC70 apoES** | **UC apoES** |
| --- | --- | --- | --- |
| ACTB | 718 | 177 | 233 |
| ANXA2 | 1032 | 347 | 192 |
| CD9 | 555 | 216 | 363 |
| CD47 | 666 | 370 | 106 |
| CD81 | 734 | 164 | 68 |
| EEF1A1 | 79 | 24 | 15 |
| ENO1 | 806 | 439 | 146 |
| FLOT1 | 206 | 84 | 75 |
| FLOT2 | 73 | 104 | 31 |
| GAPDH | 721 | 410 | 248 |
| HSPA8 | 561 | 277 | 155 |
| HSP90AA1 | 219 | 61 | 16 |
| HSP90AB1 | 65 | 12 | 35 |
| ITGB1 | 191 | 90 | 21 |
| LDHA | 195 | 85 | 110 |
| PDCD6IP | 726 | 310 | 116 |
| PKM | 686 | 366 | 149 |
| SDCBP | 1800 | 282 | 212 |
| TSG101 | 94 | 58 | 20 |
| YWHAZ | 240 | 137 | 54 |
| **Mean apoES (SD)** | **351.6 (427)** | **142.5 (138)** | **80.1 (95)** |
