# Supplementary material for: Cerebrospinal Fluid‐Derived Extracellular Vesicles: A Proteomic and Transcriptomic Comparative Analysis of Enrichment Protocols
Source: J Extracell Biol. 2025 Aug 11;4(8):e70076. doi: 10.1002/jex2.70076 (PMC12339045; doi:10.1002/jex2.70076)
Supplement: Supplementary file 7 — Supporting Table 5 RNA species identified in the output of the 3 enrichment protocols. [file JEX2-4-e70076-s002.docx]

|  | UF-SEC35 | UF-SEC70 | UC | GRCh38 |
| --- | --- | --- | --- | --- |
| Number of genes with ≥ 10 counts detected | 22440 | 18753 | 30171 | NA |
| Protein coding | 15327 | 14041 | 17440 | 19989 |
| lncRNA | 4680 | 3069 | 7980 | 20005 |
| miRNA | 18 | 13 | 37 | 1737 |
| snoRNA | 33 | 9 | 35 | *943* |
| rRNA_pseudogene | 9 | 6 | 20 | *496* |
| other | 2373 | 1615 | 4659 |  |
