# Supplementary material for: Cerebrospinal Fluid‐Derived Extracellular Vesicles: A Proteomic and Transcriptomic Comparative Analysis of Enrichment Protocols
Source: J Extracell Biol. 2025 Aug 11;4(8):e70076. doi: 10.1002/jex2.70076 (PMC12339045; doi:10.1002/jex2.70076)
Supplement: Supplementary file 8 — Supporting Table 6 Summary of the output of UF‐SEC35, UF‐SEC70, et UC enrichment protocols [file JEX2-4-e70076-s006.docx]

|  | **UF-SEC35** | **UF-SEC70** | **UC** |
| --- | --- | --- | --- |
| Mean particle size (SD) as determined by NTA in sextuplicates | 142 (3.8) nm | 150 (7.3) nm | 174 (4.7) nm |
| Mean particle yield as determined by NTA in sextuplicates | 1.09 *10^9^ | 7.42*10^8^ | 4.18*10^8^ |
| Number of identified proteins (%unique to the EV fraction) as determined by MS-DIA | 1347 (40%) | 1147 (37%) | 1624 (37%) |
| Number of genes detected with ≥ 10 counts, as determined by polyA RNA seq | 22’440 | 18’753 | 30’171 |

**Supplementary Table 6** Summary of the output of UF-SEC35, UF-SEC70, et UC enrichment protocols
